# Health in the sustainable development goals: ready for a paradigm shift?

**DOI:** 10.1186/s12992-015-0098-8

**Published:** 2015-03-21

**Authors:** Kent Buse, Sarah Hawkes

**Affiliations:** Chief, Strategic Policy Directions, UNAIDS, Geneva, Switzerland; Reader in Global Health, Institute for Global Health, University College London, London, UK

**Keywords:** Global health, Post-2015, Health policy, Evidence, SDGs, MDGs, Social determinants of health

## Abstract

The Millennium Development Goals (MDGs) galvanized attention, resources and accountability on a small number of health concerns of low- and middle-income countries with unprecedented results. The international community is presently developing a set of Sustainable Development Goals as the successor framework to the MDGs. This review examines the evidence base for the current health-related proposals in relation to disease burden and the technical and political feasibility of interventions to achieve the targets. In contrast to the MDGs, the proposed health agenda aspires to be universally applicable to all countries and is appropriately broad in encompassing both communicable and non-communicable diseases as well as emerging burdens from, among other things, road traffic accidents and pollution.

We argue that success in realizing the agenda requires a paradigm shift in the way we address global health to surmount five challenges: 1) ensuring leadership for intersectoral coherence and coordination on the structural (including social, economic, political and legal) drivers of health; 2) shifting the focus from treatment to prevention through locally-led, politically-smart approaches to a far broader agenda; 3) identifying effective means to tackle the commercial determinants of ill-health; 4) further integrating rights-based approaches; and 5) enhancing civic engagement and ensuring accountability. We are concerned that neither the international community nor the global health community truly appreciates the extent of the shift required to implement this health agenda which is a critical determinant of sustainable development.

## Introduction

Health has been recognized as central to international development for more than 20 years, and major efforts have been made to reduce morbidity and mortality either universally, or through a focus on specific population sub-groups (e.g. “the poor”, “women and children”) [1]. The eight Millennium Development Goals (MDGs), adopted in 2000, included three health-related goals to be met by 2015: reduction in child (under 5 years) mortality (Goal 4); reduction in maternal mortality and access to reproductive health care (Goal 5); and reversing the spread of HIV/AIDS, tuberculosis and malaria (Goal 6). These were instrumental in focusing global resources in low- and middle-income countries. Table 1 charts progress towards the MDG targets; we are globally on track to meet one of the three health goals (MDG 6), but although both child and maternal mortality have declined significantly, we are still not on track to reach their associated targets [2].

**Table 1:** **Progress towards the Millennium Development Goals, 2014**

**Millennium development goal**	**Targets**	**Progress on specific indicators**
**Goal 4: Reduce child mortality**	Reduce by two thirds between 1990 and 2015, the under-five mortality rate	○ Under 5 mortality decreased by 47% to date (in 2012, 6.6 million children under 5 died; majority of deaths occur in world’s poorest regions).
		○ Neonatal mortality has fallen by a third, but proportion of deaths in first 28 days of life has increased.
		○ Proportion of children covered by one dose of measles vaccine increased from 72% to 84% (from 2000–2009); no change past few years. Globally 21.2million infants unvaccinated in 2012.
**Goal 5: Improve maternal health**	Reduce by three quarters, between 1990 and 2015, the maternal mortality ratio	○ Maternal mortality ratio fell by 45% between 1990 (380 deaths per 100,000 live births) and 2012 (210 deaths per 100,000 LB). Proportion of deliveries attended by skilled health workers increased from 56% to 68% in developing countries.
	Achieve, by 2015, universal access to reproductive health	○ 83% of women in developing countries who see a health worker once in pregnancy increased to 83%, but only 52% have the 4 recommended visits.
		○ Births to adolescent girls have declined – e.g. from 88 to 50 births per 1000 girls in South Asia, but still 117 births per 1000 girls in sub-Saharan Africa, and 76 in Latin America/Caribbean.
		○ Unmet need for family planning declined from 17-12%.
Goal 6*: Combat HIV/AIDS, malaria and other diseases	Have halted by 2015 and begun to reverse the spread of HIV/AIDS	○ Number of new HIV infections (adults) declined 38% between 2001 and 2013.
	Achieve, by 2010, universal access to treatment for HIV/AIDS for all those who need it	○ In sub-Saharan Africa, 39% young men and 28% young women (aged 15–24 years) have comprehensive knowledge of HIV.
		○ 12.9 million people globally received anti-retrovirals in 2013.
	Have halted by 2015 and begun to reverse the incidence of malaria and other major diseases	○ Malaria mortality declined 42% between 2000 and 2012.
		○ 87% of 6.1 million newly diagnosed TB patients received therapy.

Data from United Nations Millennium Development Goals Report, 2014. Available at: http://www.un.org/millenniumgoals/2014%20MDG%20report/MDG%202014%20English%20web.pdf.

*HIV data from UNAIDS Global Gap Report http://www.unaids.org/en/resources/documents/2014/20140716_UNAIDS_gap_report.

In 2015, the MDGs will be superseded by the Sustainable Development Goals (SDGs). Setting global goals for health carries far-reaching and profound implications for global development. One analysis of the impact of the MDGs found that they have increased aid flows, but evidence for their impact on policy change, particularly in poorer countries, is weaker [3]. There is debate about the impact that official development assistance (ODA) for health may have on health outcomes [4]. Nonetheless, the SDGs are of significance to everyone concerned with health, justice and development as they are likely to determine the direction and level of resource commitment for global health programmes over the next 15 years in an environment where ODA plays an increasingly marginal part. Indeed the experience of the MDGs suggests that issues that do not appear in the goals are likely to receive little in the way of global (and national) attention, even if the problem is significant. For example, spending by bilateral agencies on tackling non-communicable diseases (NCDs) was lower in the late 2000s than it had been in the 1990s [5] – the MDGs excluded the NCDs despite their significant and rising proportion of the burden of global disease.

This paper outlines the process of developing the SDGs, assesses the proposed [6] health goal and targets in relation to evidence on disease burden, questions the feasibility of addressing the targets technically and politically, and reflects on the new ways of working that will be required to achieve them.

## Background

An unprecedented global consultative process emerged to develop the SDGs. Among the most prominent initiatives has been the UN Secretary-General’s High-Level Panel (HLP) on Post-2015, co-chaired by President Yudhoyono of Indonesia, President Sirleaf of Liberia, and Prime Minister Cameron of the United Kingdom, which reported in May 2013. This was informed by a series of global thematic consultations, including on health, as well as consultations in over 80 countries.

The report of the High-Level Panel was criticized as being “weak” on key aspects of global health (particularly the non-communicable diseases) [7], and questions have been raised (including by ourselves [8]) about the power of actors and decision-makers to either continue with the status quo of the MDGs, or even to actively keep “difficult” topics off the agenda. We were particularly concerned, for example, at the overt absence of any mention by the HLP of leading global health risk factors such as tobacco, alcohol and poor diet.

The report of the HLP was an intermediary step in developing the SDGs, and discussions continue to be conducted on-line, including a global poll on development priorities, [9] and through ongoing country consultations. It was in this messy process of consultations and reports that an intergovernmental Open Working Group (OWG) on SDGs was established, under the auspices of the UN General Assembly.

Co-chaired by the Ambassadors of Kenya and Hungary to the United Nations, the OWG officially consisted of 30 Member States but more than 100 countries participated as they came to see its potential importance. The Group, established at the Rio + 20 Conference in 2012, faced a challenging mandate: proposing a compelling set of goals for 2030 across the triple helix of sustainable development (economic, social and environmental) to replace the more narrow MDGs.

The OWG’s outcome document was put before the UN General Assembly in September 2014 and was adopted as the “main basis for integrating sustainable development goals into the post-2015 development agenda” [10]. The Group’s document affirms its overarching commitment to poverty eradication and breaks the agenda down into 17 goals and 126 targets, as well as listing a select set of ‘means of implementation’ (the ‘how’) under each goal. One of the 17 goals is dedicated to health, in addition progress against targets in other goals will be important to address the political, environmental, social and economic determinants of [ill-]health (Table 2). In a ‘Synthesis Report’ to the General Assembly on the SDG process at the end of 2014, the Secretary-General endorsed the OWG goals and targets [11].

**Table 2:** **Health-related targets under other goals in the Open Working Group sustainable development goal proposal**

**Proposed goal**	**Health-related targets**
**2. End hunger, achieve food security and improved nutrition, and promote sustainable agriculture**	2.1 by 2030 end hunger and ensure access by all people, in particular the poor and people in vulnerable situations including infants, to safe, nutritious and sufficient food all year round
	2.2 by 2030 end all forms of malnutrition, including achieving by 2025 the internationally agreed targets on stunting and wasting in children under five years of age, and address the nutritional needs of adolescent girls, pregnant and lactating women, and older persons
**5. Achieve gender equality and empower all women and girls**	5.2 eliminate all forms of violence against all women and girls in public and private spheres, including trafficking and sexual and other types of exploitation
	5.6 ensure universal access to sexual and reproductive health and reproductive rights as agreed in accordance with the Programme of Action of the International Conference on Population and Development and the Beijing Platform for Action and the outcome documents of their review conferences
**6. Ensure availability and sustainable management of water and sanitation for all**	6.1 by 2030, achieve universal and equitable access to safe and affordable drinking water for all
	6.2 by 2030, achieve access to adequate and equitable sanitation and hygiene for all, and end open defecation, paying special attention to the needs of women and girls and those in vulnerable situations
**11. Make cities and human settlements inclusive, safe, resilient and sustainable**	11.2 by 2030, provide access to safe, affordable, accessible and sustainable transport systems for all, improving road safety, notably by expanding public transport, with special attention to the needs of those in vulnerable situations, women, children, persons with disabilities and older persons
**16. Promote peaceful and inclusive societies for sustainable development, provide access to justice for all and build effective, accountable and inclusive institutions at all levels**	16.1 significantly reduce all forms of violence and related death rates everywhere

While some may be concerned that health has seemingly loss prominence since it has “dropped” from 3 out of 8 goals in the MDGs, to 1 out of 17 in the SDGs, we believe that this is an unwarranted concern [12]. The OWG’s single, rousing, but relatively non-specific, health goal: “*Ensure healthy lives and promote well-being for all at all ages”* is articulated through 9 targets and four mechanisms for implementation (Table 3). It is these nine targets that we review next.

**Table 3:** **Proposed means of implementation for health goal proposed by the Open Working Group**

	**Means of implementation**
1	Strengthen implementation of the Framework Convention on Tobacco Control in all countries as appropriate.
2	Support research and development of vaccines and medicines for the communicable and non-communicable diseases that primarily affect developing countries, provide access to affordable essential medicines and vaccines, in accordance with the Doha Declaration which affirms the right of developing countries to use to the full the provisions in the Trade Related Aspects of Intellectual Property Rights agreement regarding flexibilities to protect public health and, in particular, provide access to medicines for all.
3	Increase substantially health financing and the recruitment, development and training and retention of the health workforce in developing countries, especially in least developed countries and Small Island Developing States.
4	Strengthen the capacity of all countries, particularly developing countries, for early warning, risk reduction, and management of national and global health risks.

### One goal; nine targets; four means of implementation

- Target 1: *“by 2030 reduce the global maternal mortality ratio to less than 70 per 100,000 live births.”* This continues the work of MDG 5; maternal mortality has decreased substantially over the past 25 years (Table 1: *Progress towards the MDGs*), but the current global rate (210 deaths per 100,000 live births) is still off-track for reaching the target [13], and there are significant intra- and inter-country variations [2].
- Target 2: “*by 2030 end preventable deaths of newborns and under-five children.”* There has been much progress towards MDG 4, reducing deaths in children under 5. While a very large burden remains, the burden of mortality and morbidity has shifted to those infants who die in the first month of life—44% of all under-5 deaths occur at this time [14] —a group that was not specifically recognized in the MDGs [15]. The concept of “ending preventable deaths” in this population has not yet been quantified, but a *Lancet* Commission highlighted that an under-5 mortality rate of 16/1000 live births should be achievable in most low- and middle-income countries [16] (compared to the current rate in developing countries of 99/1000 live births [2]).
- Target 3: “*by 2030 end the epidemics of AIDS, tuberculosis, malaria, and neglected tropical diseases, and combat hepatitis, water-borne diseases and other communicable diseases.*” This expands substantially upon MDG 6 which only targeted AIDS, tuberculosis (TB) and malaria. Commitments to tackle water-borne diseases, for example, will tackle the 3.6% of the global burden of DALYs associated with diarrhoeal diseases - infections which are particularly burdensome in children and neonates, with half a million deaths per year in this age group [17]. Tackling the neglected tropical diseases will potentially reduce an important source of disability and chronic illness in many regions [18]. From 1990–2010 the burden of most infectious diseases fell, but Years of Life Lost (YLL, a summary measure of premature mortality) due to AIDS and TB rose substantially [19], particularly in low-income countries. The ambition to end communicable diseases is not only in line with efforts to end extreme poverty, but also backed by important political blocs, not least the African Union [20].
- Target 4: “*by 2030 reduce by one-third premature mortality from non-communicable diseases (NCDs) through prevention and treatment, and promote mental health and wellbeing.”* This new target acknowledges the fundamental shift in the global burden of disease towards the NCDs which are responsible for two of every three deaths worldwide [21]. Among the top 20 causes of YLL estimated in 2010, 7 were due to NCDs – including 2 of the top 3 (ischaemic heart disease ranks number 1 and stroke ranks third) [21]. This ambitious target also includes addressing the increasing burden of mental ill-health (depressive and anxiety disorders together account for 3.6% of the global burden of disease [18], and rates of self-harm are increasing and account for 1.5%), and the more nebulous concept of ‘well-being’ – commonly used in public discourse, but with little global agreement on its meaning, measurement or determining factors [22].
- Target 5: *“strengthen prevention and treatment of substance abuse, including narcotic drug abuse and harmful use of alcohol.”* These conditions have a significant impact on health outcomes; particularly alcohol which accounted for 2.7 million deaths and around 4% of all disability adjusted life years lost (DALYs) in 2010 [23]. However, the target addressing “substance abuse” has been given a “poor” rating by the Copenhagen Consensus, and deemed vague and/or not cost-effective [24].
- Target 6: *“by 2020 halve deaths and injuries from road traffic accidents.”* Every year an estimated 1 million men and 300,000 women die on the roads – making this the 8^th^ leading cause of death globally [14], and the 10^th^ leading contributor to DALYs [13].
- Target 7: “*by 2030 achieve universal access to sexual and reproductive health care services, including for family planning, information and education, and the integration of reproductive health into national strategies and programmes.*” The burden of disease associated with unprotected, unwanted or non-autonomous sexuality is significant: an estimated 12% of couples have an unmet need for family planning; globally there are 500 million new sexually transmitted infections every year [25]; cervical cancer is the fourth most common cause of cancer death among women worldwide [26]; and an estimated 30% of women have experienced physical and/or sexual violence from an intimate partner [27].
- Target 8: *“achieve universal health coverage (UHC), including financial risk protection, access to quality essential health care services, and access to safe, effective, quality, and affordable essential medicines and vaccines for all.”* This target represents an important addition to the agenda and has widespread backing from countries and global health institutions. The recent Ebola virus outbreak has highlighted the deficit in strong health systems coverage and access in many countries – systems that will be needed to achieve many of the other SDG targets. The ambition to shift inefficient and unjust out-of-pocket expenditure for health services to financial risk pooling arrangements aims to put an end to catastrophic payments to meet health needs which are estimated to push over 100 million people below the poverty line every year [28]. Vaccines have been identified by most analysts as a ‘best buy’ not only in global health but in development more broadly with benefits estimated at 5–15 times higher than costs [16].
- Target 9: *“by 2030 substantially reduce the number of deaths and illnesses from hazardous chemicals and air, water and soil pollution and contamination.”* In 2010, an estimated 3.5 million deaths (4.3% of global DALYs) were attributable to household air pollution, and a further 3.1 million deaths (3.1% of global DALYs) were due to ambient particulate matter air pollution [17], both ranking in the top 10 attributable risk factors for ill-health worldwide. Less is known about the global health burden associated with water and soil pollution or hazardous chemicals – including risks and intergenerational risks from the nuclear industry.

Table 3 presents four ‘means’ of implementation proposed by the OWG to support the implementation of the health agenda. These four are critical. For example, the need to take measures to train, recruit and retain health workers or strengthen the implementation of the Framework Convention on Tobacco Control. Yet, although necessary, they are probably insufficient to implement the health targets. The SDG framework includes a Goal (number 17) devoted exclusively to the Means of Implementation to support the entire agenda—which is framed as a revised global partnership—and covers the areas of finance, technology, capacity building , trade, policy coherence, partnership, data, monitoring and accountability. These were among the most contentious issues negotiated by the Open Working Group (as they relate, for example, to the responsibilities of rich nations or mechanisms to ensure accountability of the private sector) and were not resolved at the writing of this paper.

### Shifting paradigms

The OWG has proposed a comprehensive and ambitious health agenda that incorporates and builds upon the unfinished MDGs, but shifts towards a more holistic vision of health and wellbeing. While we would have configured the targets, and formulated the language of many of them differently, overall the agenda is relevant to current, emerging and predicted health burdens in the global North and South. As noted, targets are in the main focused on addressing the major burdens of illness, disability and premature mortality [29] – something that the MDGs have been criticized for overlooking [30]. Nonetheless, the OWG report raises important questions for consideration if the international community is to meet its aspiration of health as a determinant, outcome and indicator of sustainable development. In our view, meeting this aspiration will require a paradigm shift in how we approach the protection and promotion of health.

First, the framework lacks consistency in distinguishing between health sector action and action in other sectors to realize health outcomes. For example, in achieving the traffic accident and pollution targets, the health sector will play a modest role at best. Implicitly, the framework calls attention to the need for ‘health-in-all’ policies and effective collaboration and coherence across ministries and sectors – more cross-referencing across the 17 Goals and associated targets may help achieve this. In practice, success will require unprecedented inter-sectoral and intra-governmental coordination. Whether or not ministries of health, given their peripheral status in many countries, are best placed to lead such efforts is a moot point and likely varies considerably. The achievement of many health targets will require leadership from ministries other than health, which will require reforms in the health governance architecture for many countries and the way they are supported by global health institutions.

Second, as presented, the health targets may overwhelm government health sector capacity to prioritize and implement. Close reading of the goals and targets reveals that the health goal including ‘sub-areas’ contain at least 20 targets. In addition the OWG has proposed numerous health-related targets aimed at considerable disease burdens under other goals (Table 2). In contrast to the MDGs, the agenda highlights the importance of the breadth of health concerns as evidenced by the global burden of disease, but carries far-reaching implications for funding and implementation, particularly in weaker states. But it isn’t just the breadth of the challenge, but the depth. Tackling the non-communicable diseases and cutting deaths from violence or road traffic accidents will pose far more challenges than those encountered with, for example, the child or maternal survival. As we note above, ministries of health cannot carry this burden alone. But this does point to the need for doing health development differently, with much greater emphasis placed on the locally-led, globally supported and politically-smart approaches [31]. The sort of approaches that have worked with tobacco control efforts [32].

Third, in calling for significant reductions in preventable mortality from NCDs, prevention of alcohol abuse, and reducing death, disease and illness from pollution and road traffic accidents, the international community must face up to their determinants. This means addressing the commercial and other interests which stand to gain from the marketing of sugary drinks, unhealthy foods and alcohol, as well as those industries which profit from ‘dirty development’ [33]. This raises critical questions about the best approaches both nationally and globally to address ‘profit-driven diseases’ and their commercial determinants, including the potential use of sanctions against those actors who continue to market health-reducing products [34]. WHO has suggested a number of “best buys” to tackle the NCDs [35], but evidence for their implementation at national level is extremely thin. The OWG includes strengthening the implementation of The Framework Convention on Tobacco Control as one of identified means of implementation; it may provide lessons, but enactment across multiple industries will be challenging within current global health governance structures. A new approach to governing the commercial determinants of ill-health is needed [36].

Fourth, notwithstanding the reference to human rights in the preamble, there is no articulation of a rights-based approach in the health goal—not for lack of effort by many countries. While rights are important across the SDG agenda, sexual and reproductive health and rights (SRHR) constituted one of the few ‘red’ lines to some Member States during negotiations, despite the centrality of rights to enabling all individuals to achieve their sexual and reproductive health aspirations. Perhaps as a result of this, the Synthesis Report from the Secretary-General mentions only the realization of “women’s sexual and reproductive health and reproductive rights”; thus missing out on the opportunity to ensure sexual health and sexual rights for both men and women globally [37]. Rights-based approaches to health have been employed to ensure access to services and accountability in many countries (e.g. Brazil and South Africa). Such approaches, with their emphasis on social justice, non-discrimination, engagement of civil society and answerability, arguably account in large part for the unprecedented progress in many MDG areas—particularly the AIDS response [38]. We believe that achievement of the health goal will require specific efforts to elevate social justice and human rights as underlying principles for achieving sustainable health and well-being for all.

Fifth, the SDGs need to ensure a more prominent role for accountability while also promoting country ownership. Goal 17 provides a passing reference to accountability, strengthening monitoring systems and generating disaggregated data. The major investments required to monitor SDG progress should not be underestimated and the financing of the entire SDG agenda is a critical and much contested area of the international negotiations on the goals. More fundamentally, while the OWG is keen to emphasize that “countries will need to set their own targets based on national circumstances” and that “each county’s policy space” must be respected, it will be equally important that “country ownership” is not invoked at the expense of investing effort in all aspects of the agenda and leaving no one behind. Consequently, the proponents of the SDGs must recognize that progress on the MDGs was spurred in no small part by activism. In many respects it was transnational social movements which set the ‘ambition’ in the agenda (through their involvement in the international conferences leading up the Millennium Declaration) and civil society engagement in all aspects of planning and monitoring was often present where efforts succeeded—including to hold policy makers at all levels accountable for progress and results [39]. Supporting a vibrant civil society will be critical to sustainable health.

## Conclusions: implications for health post-2015

Global health is everyone’s concern, and the proposed SDGs have moved the world from a focus on the poorest countries, to an approach that is universal and equitable – in other words, the global health community should be concerned about the health of everyone, including the marginalized in middle- and high-income countries [9].

The health agenda proposed by the OWG is appropriately broad in light of current and future health needs globally—and as such represents a major improvement over the MDGs. However, its implementation demands a qualitative shift for global health as much as a quantitative one. Achieving the health goal will require leadership beyond the health sector and greater coordination across sectors. This raises the question of the extent to which the current global and national health architecture is fit-for-purpose. In our view, there are major deficiencies. In the same way that the OWG has taken the MDGs ‘out of isolation’ we now need to take the health sector out of isolation with more focus on illness prevention and promotion of wellbeing, and more collaboration with other sectors that influence health and illness outcomes—including, importantly new approaches to curb the “profit-driven” determinants of illness.

Implementation will require a great deal of new investment. Now is the time for the global health community to clearly articulate the well-established returns on investments in health—reaffirmed in The Lancet Commission’s ‘Investing in Health 2035’ [16]. The OWG calls to “increase substantially health financing.” Resources will invariably be constrained. Yet the new goal and targets may be achievable if we get real about prevention rather than relying on the fallback of treatment. Given the prohibitive costs of curative and chronic treatments—consider the price tag of USD 84,000 for a full course of Hepatitis C treatment (one of the OWG targets from which over 100 million people could benefit [40]) – we may have no choice but to push for prevention.

For the major global causes of premature death and disability (particularly heart disease, chronic obstructive pulmonary disease, diabetes, lung cancer), prevention will require fundamentally rethinking how we approach the commercial determinants of illness and “profit driven diseases”. It will also demand a rethink of our approach to the development and pricing of vaccines and drugs. The OWG calls for “support” for R&D and access to “affordable” medicines and vaccines—and in this regard for the full implementation of TRIPs flexibilities. Yet as others have pointed out, R&D will require fundamentally new models which are not solely profit-oriented [41]. The OWG recognizes the need for stepped up “recruitment, development and training and retention of the health workforce in developing countries, especially in LDCs and SIDS.” But in addition to quantity, it is arguably the case that the health workforce itself needs to be retooled and brought closer to communities if health-as-a-way-of-life is to be achieved [42].

The SDG process offers an opportunity to reimagine global health and its centrality to sustainable development. The OWG proposal provides a useful guide to what we ought to aspire to achieve. We must now offer a credible game plan on how to deliver it so as to advance human dignity, equity and sustained well-being. In our view, this will require nothing less than a paradigm shift in global health.

## Abbreviations

DALYs: Disability adjusted life year
LDCs: Least developed countries
MDGs: Millennium development goals
NCDs: Non-communicable diseases
OWG: Open working group
R&D: Research and development
SDGs: Sustainable development goals
SIDS: Small island developing states
YLL: Years of life lost
